# Post-discharge depressive symptoms and health-related quality of life of critical COVID-19 survivors

**DOI:** 10.1192/j.eurpsy.2021.692

**Published:** 2021-08-13

**Authors:** S. Martins, A.R. Ferreira, J. Fernandes, T. Vieira, L. Fontes, I. Coimbra, J. Paiva, L. Fernandes

**Affiliations:** 1 Department Of Clinical Neuroscience And Mental Health And Center for health technology and services research (cintesis), Faculty of Medicine - University Porto, Porto, Portugal; 2 Intensive Care Medicine Department, Centro Hospitalar Universitário São João (CHUSJ), Porto, Portugal; 3 Department Of Medicine, Faculty of Medicine - University Porto, Porto, Portugal; 4 Psychiatry Service, Centro Hospitalar Universitário São João (CHUSJ), Porto, Portugal

**Keywords:** COVID-19, ICU survivors, Depression, quality of life

## Abstract

**Introduction:**

Survivors of critical illness stay frequently experience long-term mental health morbidity, suggesting that many critically ill patients with COVID-19 may also show a high prevalence of psychiatric conditions.

**Objectives:**

To describe depression in COVID-19 survivors 4-months post-hospital discharge and to examine its association with health-related quality of life (HRQoL).

**Methods:**

This pilot study involved COVID-19 adult patients admitted in Intensive Care Medicine Service (ICMS) of a University Hospital. Exclusion criteria were: ICMS length of stay (LoS)≤24h, terminal illness, major sensory loss and inability to communicate at the time of assessment. All participants were evaluated at ICMS scheduled telephone follow-up appointment, with Patient Health Questionnaire (PHQ-9) (depression) and EQ-5D-5L (HRQoL). Critical-illness severity was assessed with APACHE-II and SAPS-II.

**Results:**

Twenty patients were included with a median age of 62(range: 24-77) y.o., the majority male (75%) and married (70%). Median (range) APACHE-II and SAPS-II was 17 (5-34) and 32.5 (7-77), respectively, and LoS was 18 (4-58) days. Overall, 25% patients presented depression symptoms and most reported problems on EQ-5D-5L domains of pain/discomfort (65%), anxiety/depression (55%) and mobility (50%). Depression scores were higher in patients with problems in EQ-5D-5L domains of usual activities (median 4 vs 1.5; p=0.046), pain/discomfort (median 0 vs 4; p=0.004) and anxiety/depression (median 4 vs 0;p<0.001).

**Conclusions:**

These preliminary findings show that depression is frequent in COVID-19 survivors and it is associated with worse HRQoL. This pilot study highlights the importance of psychological assessment and treatment of COVID-19 survivors, in order to minimize its negative impact on HRQoL, optimizing their recovery.

